# Genome Wide Association Analysis Reveals New Production Trait Genes in a Male Duroc Population

**DOI:** 10.1371/journal.pone.0139207

**Published:** 2015-09-29

**Authors:** Kejun Wang, Dewu Liu, Jules Hernandez-Sanchez, Jie Chen, Chengkun Liu, Zhenfang Wu, Meiying Fang, Ning Li

**Affiliations:** 1 Department of Animal Genetics and Breeding, National Engineering Laboratory for Animal Breeding, MOA Laboratory of Animal Genetics and Breeding, College of Animal Science and Technology, China Agricultural University, Beijing, 100193, People’s Republic of China; 2 Guangdong Provincial Key Lab of Agro-Animal Genomics and Molecular Breeding, College of Animal Science, South China Agricultural University, Guangzhou, Guangdong, 510642, People’s Republic of China; 3 Research Methods Group| Institute of Health and Biomedical Innovation (IHBI), Queensland University of Technology (QUT), 60 Musk Ave/cnr. Blamey St, Kelvin Grove, QLD 4059, Australia; 4 State Key Laboratory for Agrobiotechnology, China Agricultural University, Beijing, 100094, People’s Republic of China; China Agricultural Univeristy, CHINA

## Abstract

In this study, 796 male Duroc pigs were used to identify genomic regions controlling growth traits. Three production traits were studied: food conversion ratio, days to 100 KG, and average daily gain, using a panel of 39,436 single nucleotide polymorphisms. In total, we detected 11 genome-wide and 162 chromosome-wide single nucleotide polymorphism trait associations. The Gene ontology analysis identified 14 candidate genes close to significant single nucleotide polymorphisms, with growth-related functions: six for days to 100 KG (WT1, FBXO3, DOCK7, PPP3CA, AGPAT9, and NKX6-1), seven for food conversion ratio (MAP2, TBX15, IVL, ARL15, CPS1, VWC2L, and VAV3), and one for average daily gain (COL27A1). Gene ontology analysis indicated that most of the candidate genes are involved in muscle, fat, bone or nervous system development, nutrient absorption, and metabolism, which are all either directly or indirectly related to growth traits in pigs. Additionally, we found four haplotype blocks composed of suggestive single nucleotide polymorphisms located in the growth trait-related quantitative trait loci and further narrowed down the ranges, the largest of which decreased by ~60 Mb. Hence, our results could be used to improve pig production traits by increasing the frequency of favorable alleles via artificial selection.

## Introduction

The pig is an important farm animal worldwide, providing over ~37% of all meat average during the year 2012–2014 (http://www.fao.org/ag/againfo/themes/en/meat/background.html). Efficient meat production is paramount in livestock and there is an expected positive correlation between meat production and growth rate [1]. Average daily gain (ADG), days to 100KG (D100) and Feed conversion ratio (FCR) are considered as target traits to measure the growth rate and production performance. Therefore, understanding the genetic determinants controlling FCR, D100 and ADG is crucial for designing better breeding programs and improving production efficiency.

13030 QTLs from 477 publications were reported to be associated with 663 different pig traits [2]. 1424 QTLs are associated with production traits, which including 312 QTLs for ADG; 12 QTLs for days to different body weight; 93 QTLs for FCR (http://www.animalgenome.org/cgi-bin/QTLdb/SS/index, Apr 27, 2015). However, even though there are some successful examples of QTLs found in domestic animals [3, 4], identification of causative mutations underlying QTLs is still a challenge [5]. Poor resolution in QTL mapping experiments (i.e. large region in genome consist of hundreds or thousands of genes) and complicated architecture in most QTLs (i.e. multiple causative mutation present in one or several genes) make QTLs mapping not very successful [5]. Moreover, QTLs are inconsistently replicated in different source populations [6].

GWAS is well-known and powerful strategy for genetic dissection of trait loci in human and animal due to the development of high throughput SNP platform and cost-effective method for large population analysis. Furthermore, it is believed that GWAS signals have replicated across populations of different regions [7] and was proved by some reported researches [8–10]. Recent technological advances, such as the complete pig genome sequence and the 60K porcine SNP chip array, have facilitated genome-wide association studies (GWAS) in this species [11]. Several GWA analysis has been performed for searching production trait-related candidate genes in varied pig populations [12–16]. 127 significant SNPs (*P*_Bonferroni_ <0.01) and 102 suggestive SNPs (*P*_Bonferroni_ <0.10) were detected for ADG in two extreme and divergent groups of Italian Large White pigs [12]. Another GWAS study was implemented within two extremely divergent purebred Yorkshires lines, their results showed that significant SNPs for residual feed intake and ADG were identified on different chromosomes (SSC3, SSC5, SSC6, SSC7, SSC13, SSC14, and SSC15) [16]. Duroc is an excellent source of sires for pig production, it is important to find out growth-related potential genes for molecular breeding. However, only one GWAS were carried out in this breed, in total 110 significant SNPs were detected for FCR [13]. In this study, we perform GWAS for D100, FCR, and ADG using Illumina Porcine SNP60 BeadChip in a ~800 male Duroc pig population to understand the genetic mechanisms underlying such important traits.

## Materials and Methods

### Source population and phenotypes

A total of 796 commercial Duroc sires from the Guangdong Wen’s Foodstuffs Group Co., Ltd. (Guangdong, China) were used in this study. All animals were born at the end of 2011, and raised in the same standard conditions and no open wounds of other signs of illness of injury and no display abnormal behavior etc. Ear tissue collection was implemented based on the procedure below: pig ear was first cleaned with 75% alcohol followed by cutting the small fraction of ear with clear forfex, and then treating the wound with tincture of iodine. The protocol for ear tissue collection was approved by the Animal Welfare Committee of the China Agricultural University (approval number: XK257).

Traits recorded for individual boars are D100 (Days to 100 KG of body weight), FCR (feed conversion ratio between 30 and 100 KG), and ADG (average daily gain between 30 and 100 KG). Phenotypes were collected by Osborne FIRE Pig Performance Testing System (Kansas, American) in Guangdong Wen’s Foodstuffs Group Co., Ltd. (Guangdong, China). ADG and FCR was tested between 30 KG and 100 KG of body weight. D100 was measured from birth to 100 KG of body weight.

### Genotyping and quality control

DNA was extracted from ear tissue using the phenol-chloroform method [17]. The quality and quantity of the DNA extracted was checked with a NanoDrop™ 2000 (Thermo Fisher Scientific Inc., USA). DNA quality was measured by retaining samples with concentrations >50 ng/μl, total volume >50 μl, and a ratio of light absorption (A260/280) between 1.8 and 2.0. Genotyping was conducted using the Illumina Porcine SNP60 BeadChip by the company (DNA LandMarkers, Canada) Genotypes were called with GenomeStudio (Illumina, USA). Data mining was performed in our lab.

To reduce the false-positive associations resulting from genotyping, we controlled our SNP analysis with a genotyping call rate ≥ 95% and a Hardy–Weinberg equilibrium (HWE) p ≥ 10^−4^. Considering that rare SNPs have lower statistical power, SNPs with a minor allele frequency (MAF) ≥ 1% were selected for further analysis. Moreover, all of the SNPs located on the sex chromosome were removed.

### GWAS and population stratification assay

Genome-wide association studies were performed by testing the association for each SNP-trait combination independently. The potential bias in association caused by hidden population structures was removed by adjusting phenotypes and genotypes as suggested by Price et al. [18]. We used the EGSCORE function (EIGENSTRAT method) in the GenABEL R package [19]. Via EIGENSTRAT method, the genotypes and phenotypes were corrected by regressing them onto principal axes of variation obtained by decomposing the identity-by-state (IBS) matrix among individuals [18]. Then the association between the ancestry-adjusted phenotype values and each ancestry-adjusted SNP was computed with a linear regression model. The quantile–quantile (Q–Q) plot was always implemented in the test, this is a commonly used tool for scanning the population stratification in GWA studies [20]. Multiple testing was carried out for permutations while GenABEL/egscore function was performed with times = 10,000 argument [19]. The permutation at genome-wise significance or chromosome-wise significance was implemented with all filtered SNPs in the whole genome or a particular chromosome [21]. The phenotypes of three traits were randomly shuffled 10,000 times and the empirical threshold value for genome-wise and chromosome-wise was determined by selecting the 95th percentile of the highest test statistic over the 10,000 permutation replicates [22, 23]. An adjusted p-value for each SNP were obtained after permutation, and then we defined a SNP is genome-wide significant (significant) or chromosome-wide significant (suggestive) if its adjusted p-value is less than 0.05 [24].

### Haplotype block analysis

Whole genome haplotype block was estimated by PLINK software [25], with the default Haploview procedure. Haplotype block analysis was implemented within chromosomes with at least two significant SNPs. The haplotype blocks were defined by the criteria of Gabriel et al. to further pinpoint underlying associations affecting the trait [26, 27].

### Gene ontology analysis

Genomic locations for the Sscrofa 10.2 genome version were downloaded from www.animalgenome.org/pig/. The SNP linkage map is based on USDA-MARC v2 (A) (http://www.thearkdb.org/). Selection of the nearest gene to the significant SNPs was obtained from www.ensembl.org/Sus_scrofa/Info/Index (Sscrofa 10.2 genome version). To obtain the closest human homology genes in the gene list, we input the pig gene ID into the Ensemble BioMart (http://www.ensembl.org/biomart/martview). Gene ontology analysis was carried out using the DAVID Bioinformatics Resources 6.7 (http://david.abcc.ncifcrf.gov/) [28].

## Results

### Phenotype and SNP data summary

Phenotype data of three production traits were analyzed and presented in Table 1. All traits were approximately normally distributed. After quality controlled filtering steps, 39,436 SNPs were available for GWA analysis (Table 2). The average physical distance between two neighboring SNPs on the same chromosome was approximately 56.7Kb, ranging from 48.3 (SSC10) to 76.4 Kb (SSC1). Based on the length of each chromosome in the USDA-MARC v2 (A) linkage map, the average genetic distance between adjacent SNPs on the SNP chip was 0.062 cM, this ranged from 0.096 cM (SSC12) down to 0.037 cM (SSC1) (Table 2). A comparison of different SNP chip found, the higher density (shorter average distance) between adjacent SNPs, the finer genomic region will be obtained for GWAS and haplotype block analysis.

**Table 1:** Descriptive statistics analysis of production traits in a male Duroc population.

Traits	Units	N	Mean	SD	Min	max
**D100**	day	792	161.773	8.091	136.6	196.9
**ADG**	g/day	792	868.37	100.625	547.5	1211.49
**FCR**	kg/kg	790	2.108	0.175	1.59	2.76

Mean, standard deviation (SD), minimum (min) and maximum (max) values are presented for all of the phenotypes included in the association study (N).

**Table 2:** Distributions of SNPs after quality control and the average distance between adjacent SNPs on each chromosome.

SSC	SNP no.	Physical size (Mb)^1^	Mb/SNP	Linkage map (cM)^2^	cM/SNP
**1**	3866	295.5	0.0764	144	0.0372
**2**	2505	140.1	0.0559	132	0.0527
**3**	1972	123.6	0.0627	129	0.0654
**4**	2581	136.3	0.0528	130	0.0504
**5**	1580	100.5	0.0636	114	0.0722
**6**	2160	123.3	0.0571	165	0.0764
**7**	2302	136.4	0.0593	156	0.0678
**8**	2258	120	0.0531	127	0.0562
**9**	2237	132.5	0.0592	138	0.0617
**10**	1381	66.74	0.0483	124	0.0898
**11**	1486	79.82	0.0537	85	0.0572
**12**	1180	57.44	0.0487	113	0.0958
**13**	2821	145.2	0.0515	126	0.0447
**14**	2848	148.5	0.0521	111	0.0390
**15**	2162	134.5	0.0622	112	0.0518
**16**	1416	77.44	0.0547	93	0.0657
**17**	1268	64.4	0.0508	97	0.0765
**18**	935	54.31	0.0581	57	0.0610
**Unmapped**	2478				
	39436	2136.55			

SNP, single nucleotide polymorphisms; SSC, Sus scrofa chromosome

^1^The physical size is based on Sus scrofa Build 9 (http://www.ensembl.org/Sus_scrofa/Info/Index)

^2^The linkage map is based on USDA-MARC v2 (A) (http://www.thearkdb.org/).

### Significant SNPs and phenotypic variance

**Fig 1:**
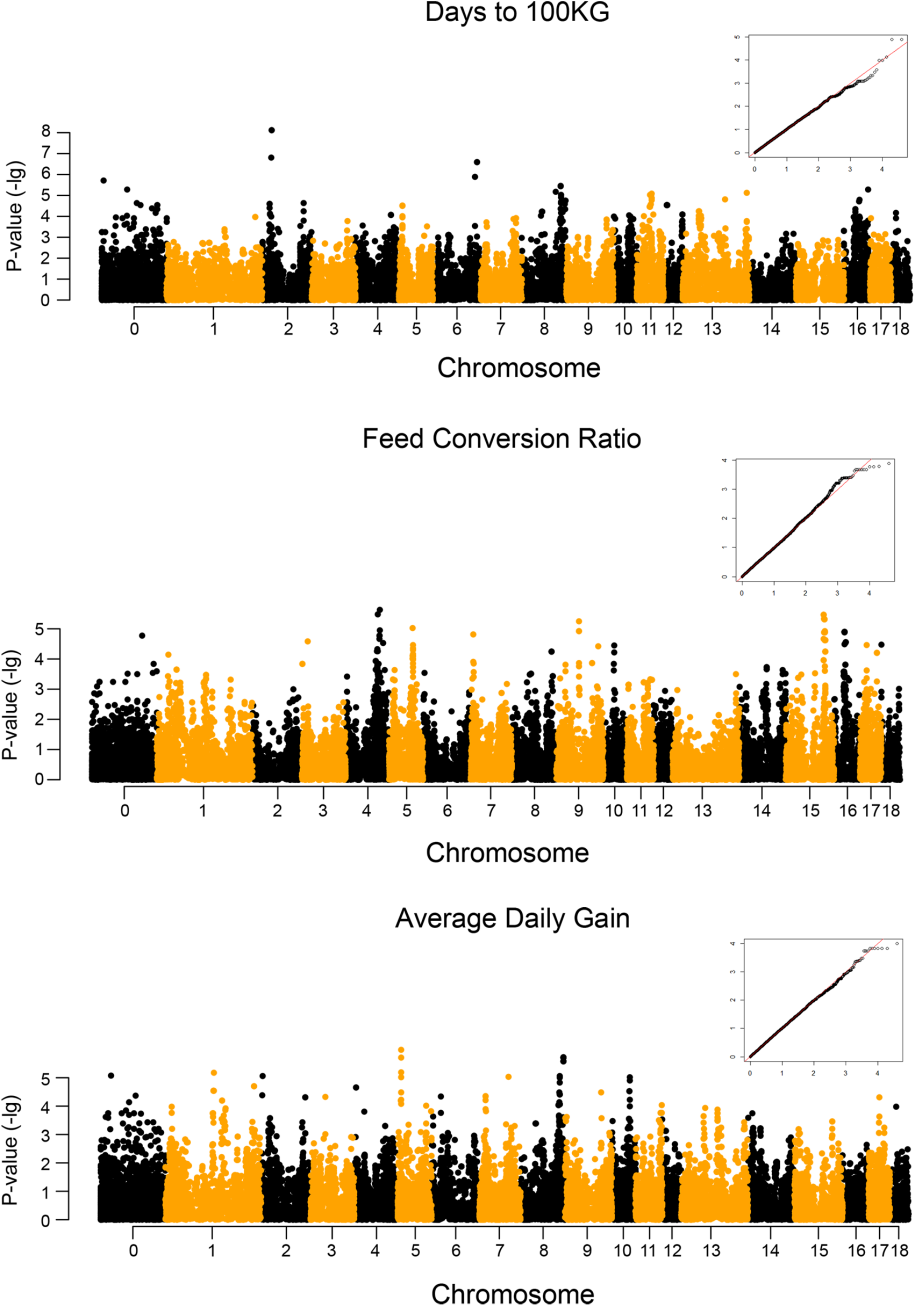
Manhattan plots of genome-wide association studies for three production traits in male Duroc pigs. The inserted quantile–quantile (Q–Q) plots show the observed versus expected log p-values.

The p-value of (in terms of–log_10_ p) profiles of all SNPs association tested for the three traits examined are shown in Fig 1. The genome-wide significant SNPs at the permutation based critical level detected by the associated test for the three traits are shown in Table 3. In total, 11 genome-wide significant (significant) and 162 chromosome-wide significant (suggestive) SNPs were defined. The proportion of phenotypic variance explained by each significant SNP is shown in Table 3.

**Table 3:** Genome-wide significant SNPs and closest genes for D100 and FCR traits.

Trait	No.	SNP ID	SSC^1^.	Location (bp)^2^	Adjusted p-value	Phenotypic variance explained by SNP (%)	Nearest gene	Distance/bp	Relative function
**D100**	7	M1GA0027152	0	0	0.032	2.86	NA	NA	NA
		MARC0024404	2	30918580	2.0E-04	4.21	WT1	within	DNA binding
		MARC0041999	2	30971975	2.0E-04	4.21	ENSSSCG00000024950	(+)15767	NA
		DRGA0002876	2	29530558	2.9E-3	3.48	FBXO3	within	protein ubiquitination
		ALGA0110196	6	144977414	5.7E-3	3.35	ENSSSCG00000024345	(+)289423	NA
		ASGA0030028	6	144900841	5.7E-3	3.35	ENSSSCG00000024345	(+)365996	NA
		MARC0091155	6	138154672	0.0213	2.96	DOCK7	within	neuron development
**FCR**	4	INRA0016084	4	112040937	0.0313	2.82	TBX15	(-)5882	transcription factor activity
		INRA0015807	4	105568818	0.044	2.74	IVL	(+)28421	structural molecule activity
		ALGA0086784	15	123675955	0.0451	2.73	ENSSSCG00000023447	(-)93555	NA
		ALGA0086789	15	123902773	0.0451	2.73	MAP2	(+)201050	phosphorus metabolic process

SNP, single nucleotide polymorphisms; D100, days to 100 KG; FCR, feed conversion ratio; WT1, Wilms’ tumor 1; FBXO3, F-box only protein 3; DOCK7, Dedicator of cytokinesis 7; TBX15, T-box 15; IVL, involucrin; MAP2, microtubule-associated protein 2

^1^Sus scrofa chromosome

^2^Derived from the current porcine genome sequence assembly (Sscrofa10.2) (http://www.ensembl.org/Sus_scrofa/Info/Index)

+/-: The SNP located in the upstream/downstream of the nearest gene; NA: not assigned.

Regarding D100, seven significant SNPs were detected (Table 3). One SNP (*M1GA0027152*) had no known location; the remaining significant SNPs were located on SSC2 and SSC6. Of these, five SNPs reached the 1% genome-wide significance (adjusted p-value < 0.01) level. Moreover, 78 suggestive SNPs were detected; these were mainly located on SSC2, SSC8, SSC11, SSC12, and SSC16 (S1 Table). Twenty-six suggestive SNPs involved in the D100 trait were located in the interior regions of known genes in the Ensemble Sscrofa 10.2 assembly. The nearest genes for the remaining mapped SNPs are shown in S1 Table.

For FCR, four significant SNPs were found, of which two were located on SSC4 and two on SSC15 (Table 3). Additionally, of the remaining 66 suggestive SNPs most were located on SSC4 (n = 8), SSC15 (n = 24), and SSC16 (n = 14) (S1 Table). Twenty-four suggestive SNPs were identified in the inner regions of known genes.

However, the permutation tests revealed no significant association for ADG. Only 24 suggestive SNPs were detected and most were located on SSC8 (n = 9) and SSC10 (n = 6) (S1 Table). Among these SNPs, nine were located within genes. Several suggestive SNPs were associated with more than one trait, indicating possible pleiotropic effects. For example, nine suggestive SNPs were associated with both D100 and ADG on SSC8.

### Candidate genes at significant or suggestive level

The aim of this study was to identify and characterize novel growth-related genes in the pig. After obtaining the above results, we tried to reduce the number of potential genes based on a common growth-related biological function. A list of 14 candidate genes was obtained. Of these, Wilms’ tumor 1 (WT1), F-box only protein 3 (FBXO3), Dedicator of cytokinesis 7 (DOCK7), Protein phosphatase 3, catalytic subunit, alpha isozyme (PPP3CA), 1-acylglycerol-3-phosphate O-acyltransferase 9 (AGPAT9), and NK6 homeobox 1 (NKX6-1) associated to D100; microtubule-associated protein 2 (MAP2), T-box 15 (TBX15), involucrin (IVL), ADP-ribosylation factor-like 15 (ARL15), carbamoyl-phosphate synthase 1, mitochondrial (CPS1), von Willebrand factor C domain-containing protein 2-Like (VWC2L), and VAV3 guanine nucleotide exchange factor (VAV3) correlated with FCR; collagen, type XXVII, and alpha 1 (COL27A1) as a potential functional candidate gene, associated to ADG. Gene ontology analysis indicated that most of the candidate genes are involved in muscle, fat, bone or nervous system development, nutrient absorption, and metabolism.

### Population stratification

**Fig 2:**
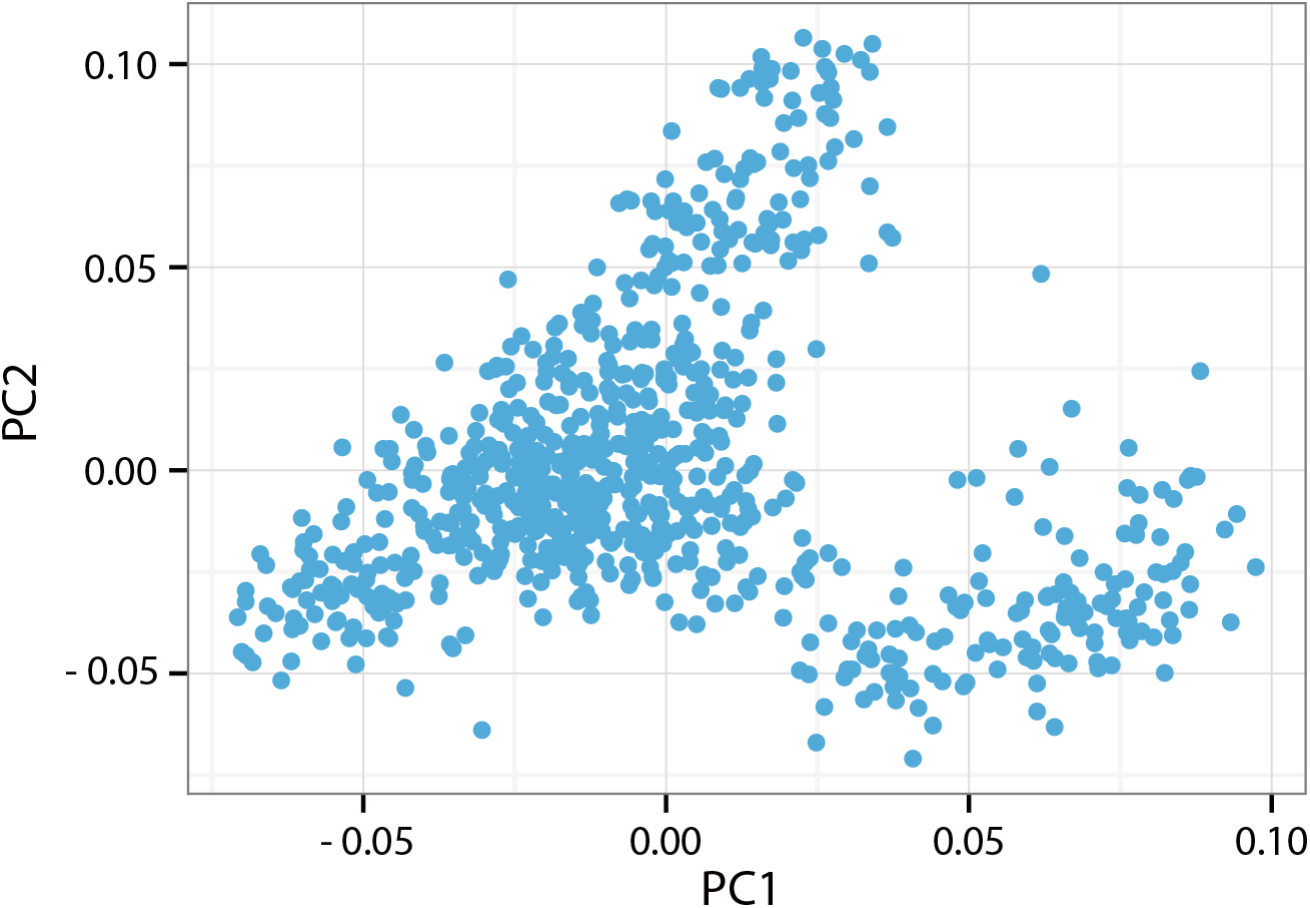
Principle component analysis (PCA) plot of population structure with the top two principle components. PC1: Principle component 1; PC2: Principle component 2.

**Fig 3:**
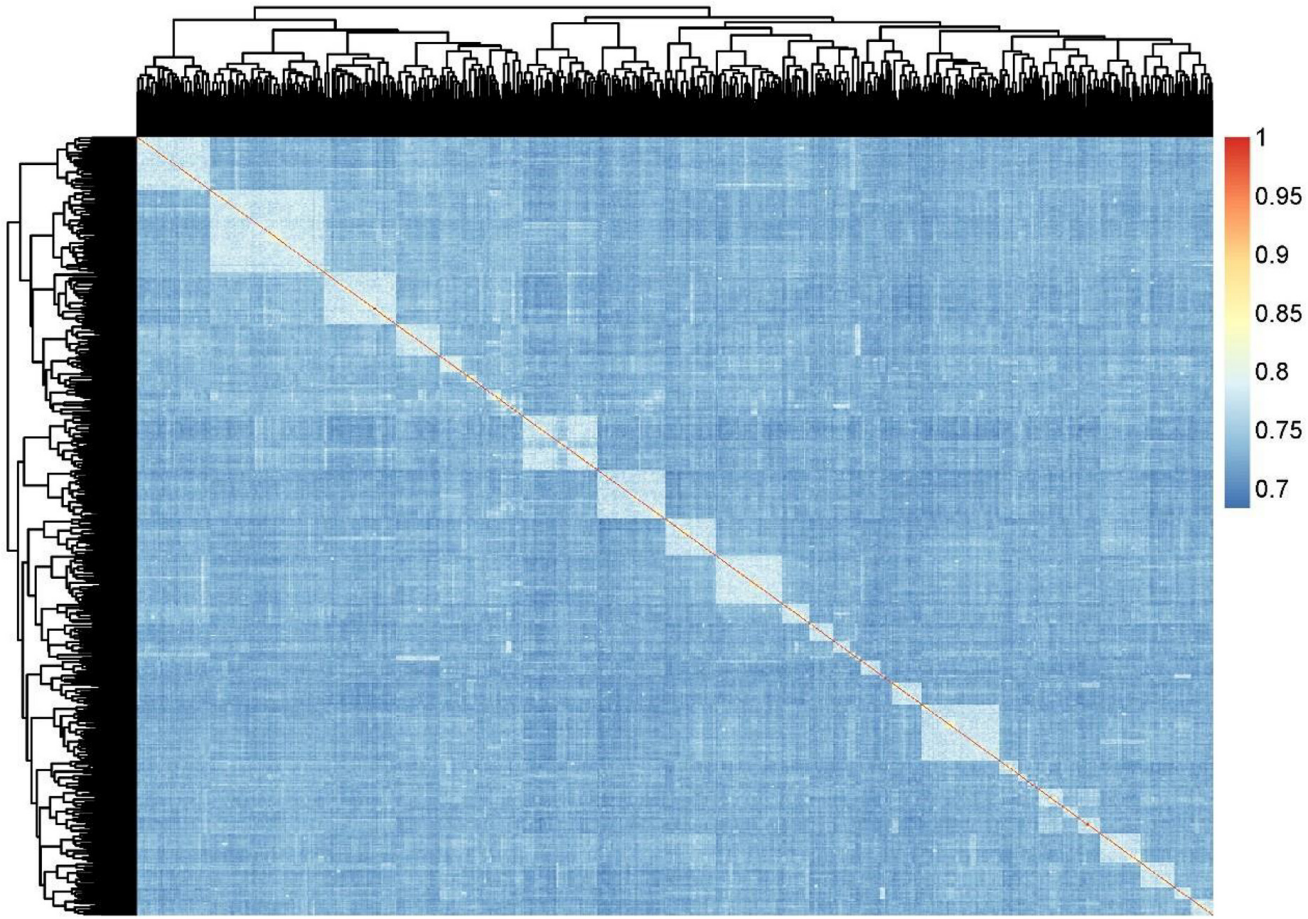
IBS similarity matrix.

The power of genetic association analysis is often compromised by population stratification, which contributes to false positive results. To investigate the population structure, we constructed a principle component analysis (PCA) analysis and plotted the filtered SNP data with first two principle components (Fig 2). The contribution rate of the first two principle components (Principle component 1 and Principle component 2) were 2.78% and 2.31% respectively and the cumulative contribution rate of top ten principle components were 18.25% (S1 Fig). Our further analysis, based on the IBS status, also gave us a similar population structure (Fig 3). We adjusted our data to prevent false positive signals from stratification, even though the evidence for population stratification was not strong.

### Haplotype block analysis

**Fig 4:**
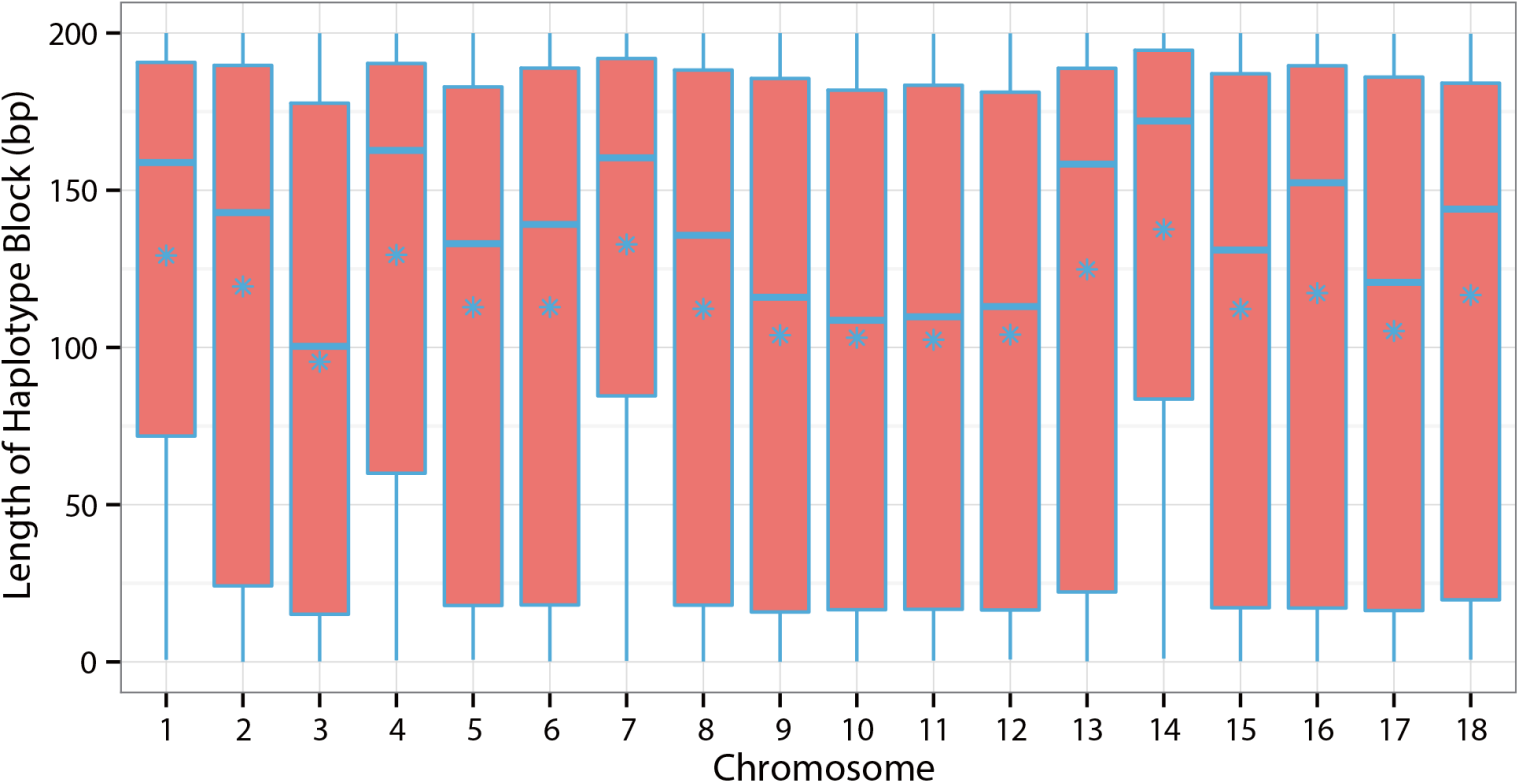
Distribution of haplotype length along the genome. *denotes mean length.

**Fig 5:**
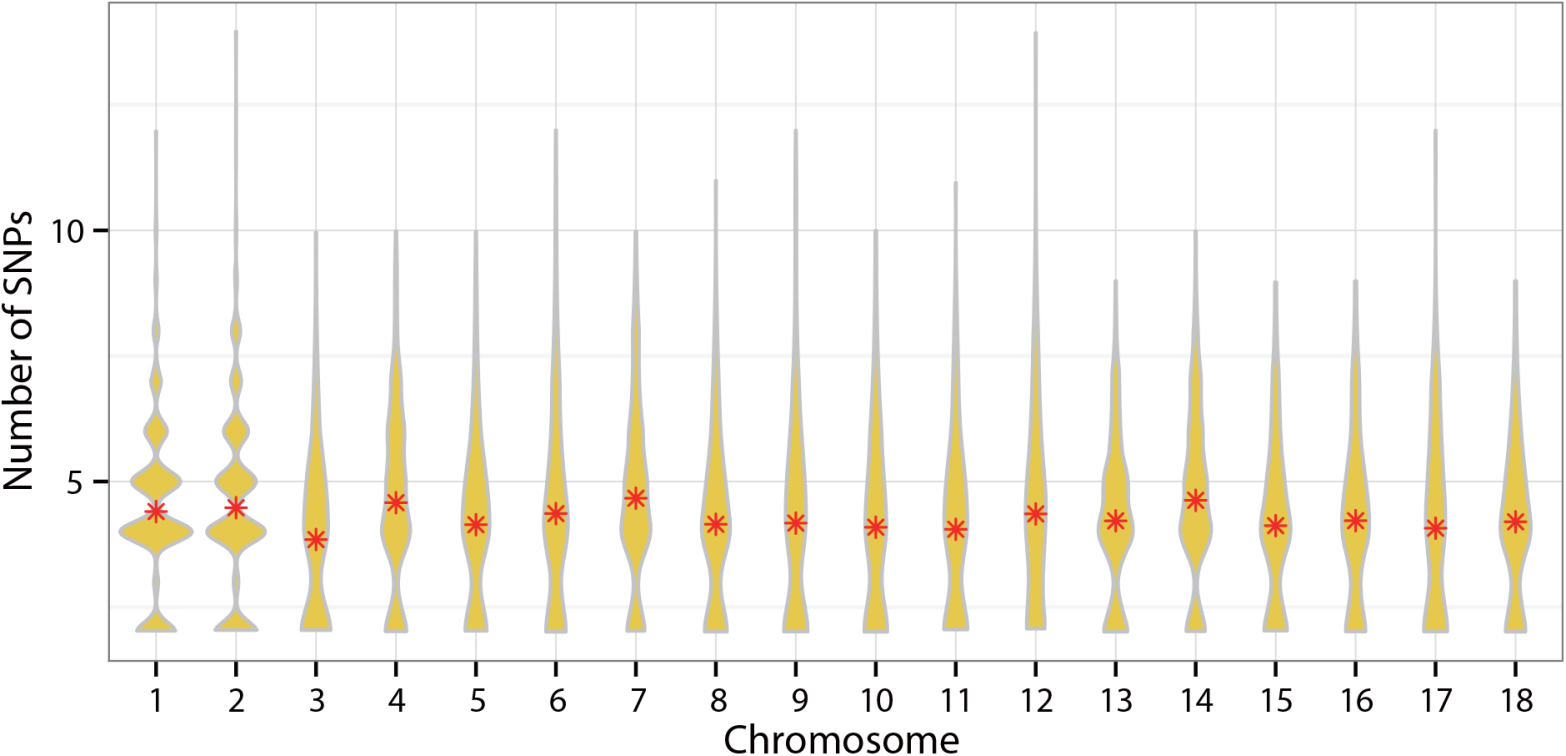
Distribution of the number of SNPs in each haplotype block along the genome. *denotes mean number of SNPs.

**Fig 6:**
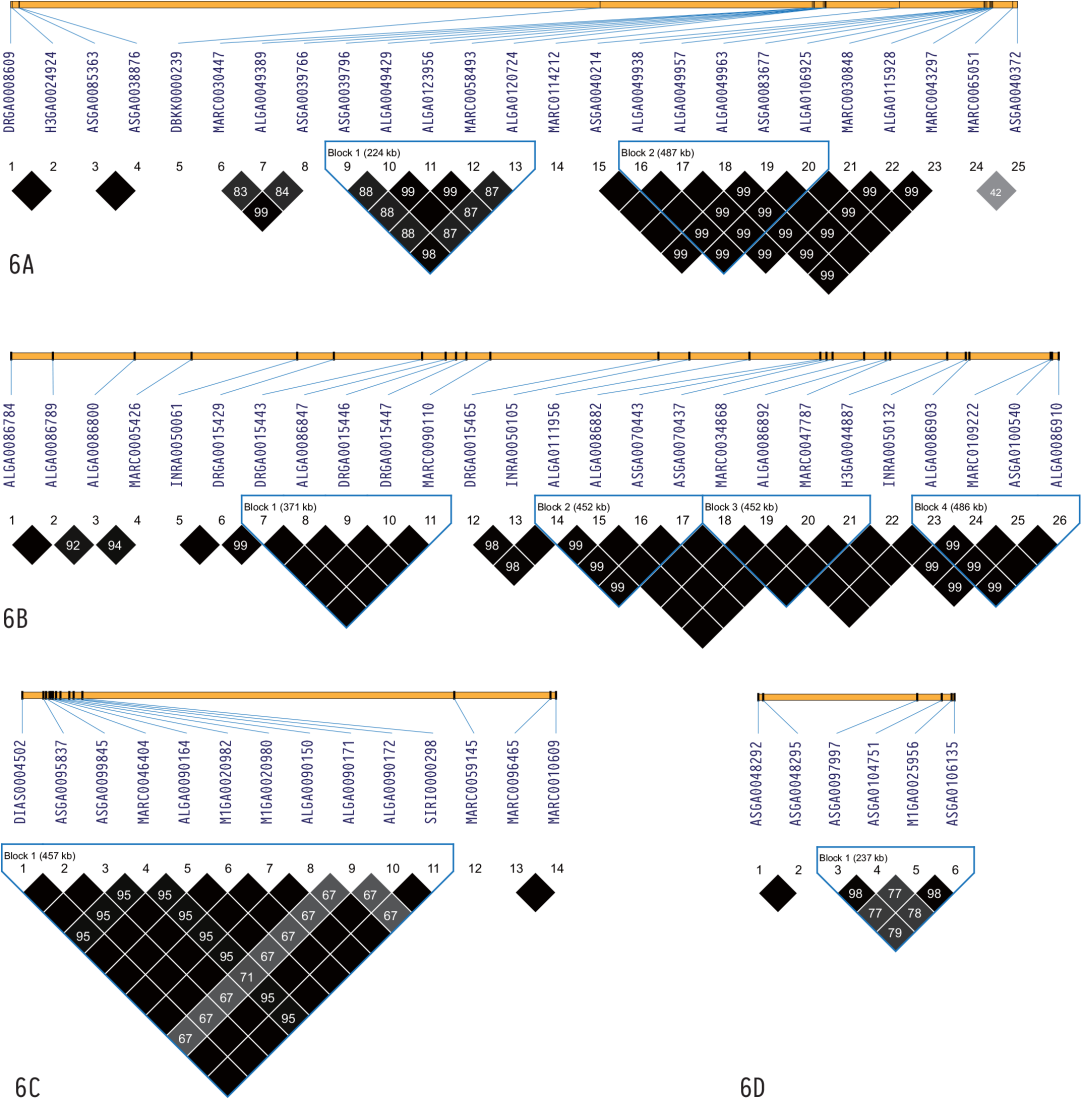
Haplotype blocks for significant SNPs. The black line indicated the identified blocks. 6A: A haplotype block composed of suggestive D100 SNPs located in SSC8; 6B: A haplotype block composed of suggestive FCR SNPs located in SSC15; 6C: A haplotype block composed of suggestive FCR SNPs located in SSC16; 6D: A haplotype block composed of suggestive ADG SNPs located in SSC10.

In total, 4,975 haplotype blocks were obtained in our study. The number of SNPs ranged from 2 to 14. The average haplotype block length was 117.1559 Kb and the longest was 199.999 Kb (Fig 4). The distribution of haplotype length and the number of the SNPs are shown in Figs 4 and 5. However, the haplotype blocks were not distributed evenly on all chromosomes. In our study of D100, we found 25 suggestive SNPs located in SSC8 from 67.4 to 144.2 Mb and 2 strong haplotype blocks were detected (129.4–129.6 Mb and 141.7–142.2 Mb) (Fig 6A). Five genes, DDIT4L, H2AFZ, PTPN13, MAPK10, and ARHGAP24, were located in the two blocks (S1 Table). In our study of FCR, 26 suggestive SNPs located in SSC15 ranging from 123.7 to 129.4 Mb were detected and they constituted strong haplotype blocks (125.9–126.3 Mb, 127.7–128.1 Mb, 128.3–128.8 Mb and 128.9–129.4 Mb) (Fig 6B). Genes including ERBB4, IKZF2, SPAG16, VWC2L, ENSSSCG00000029683, and ENSSSCG00000029020 were identified in this region (S1 Table). We also observed 11 suggestive FCR SNPs located in SSC16 from 34.9 to 38.9 Mb, of which 10 SNPs were located at 35 Mb. A particularly strong haplotype block from 34.85 to 35.31 Mb was identified in our data (Fig 6C). Further analysis found that the ARL15, NDUFS4, and ENSSSCG00000024947 genes were located in this haplotype block (S1 Table). As for ADG, however, relatively few suggestive SNPs generated less haplotype blocks. It is important to mention that a haplotype block located in SSC10 from 59.0 to 59.2 Mb contained the genes MLLT10 and SKIDA1 (Fig 6D) (S1 Table).

## Discussion

Duroc pig is an excellent source of sires for pig production, and is particularly crucial to the improvement of growth and lean meat traits of pig populations. Thus, it is important to obtain major genes responsible for growth traits for future molecular breeding. GWAS provides an efficient way to search for growth-related candidate genes in Duroc pigs. We already know that the accuracy of GWAS and haplotype block analysis is based on the population structure, such as genome-wide linkage disequilibrium extent [29]. Compared with human population, domestic animals have simpler population structure and genetic diversity, especially within one breed [5]. So, GWAS is more useful for domestic animals, including pig. In this study, all Duroc pig samples were collected from the same farm, and could be treated as having a similar genetic background, which is verified by PCA clustering and IBS status results (Figs 2 and 3). The investigated pigs without population stratification was also confirmed by Q–Q plots (Fig 1), which showed the obtained results is not deviate from expected values. Therefore GWAS became the method of choice in this analysis.

Candidate gene searches following GWAS consist of descriptions of genes located close to significant SNPs that are physiologically related to the traits of interest. Thus, potentially important but apparently physiologically unrelated genes may be discarded from further analysis. In this study, given that we are interested in the genetics of fast and efficient growth, we expected to find genes involved in fat, muscle, bone or nervous tissue development, cell proliferation and differentiation, nutrient absorption, and metabolism. Then, 14 candidate genes, located close to significant or suggestive SNPs were considered as important candidate genes.

Six candidate genes associated with D100 trait were selected. The WT1 gene has a crucial role in organ development from cell proliferation to mature organ structure [30]. Little is known about the FBXO3 and DOCK7 genes, although they are located near the most significant SNPs. Three suggestive SNPs were located in the PPP3CA intron and another two were located in the AGPAT9 and NKX6-1 introns. PPP3CA activates myogenin gene transcription [31]. In transgenic mice overexpressing PPP3CA, glucose absorption and glycogen and lipid oxidation in skeletal muscle increased [32]. Furthermore, AGPAT9 is a member of the GPAT gene family that controls the rate of triacylglycerol biosynthesis [33]. NKX6-1 is active in developing pancreatic β-cells [34].

We selected seven candidate genes associated with FCR. The MAP2 gene has a role in neuron growth and repair [35]. It was reported that body weight was regulated through the central nervous system, because glucocorticoid and mineralocorticoid receptors in hippocampal neurons define the balance between glucose allocation processes and food intake [36]. The TBX15 gene is involved in adipocyte differentiation, triglyceride accumulation, and mitochondrial function, and some of its variants reportedly increase the risk of diabetes and metabolic disease [37]. IVL, a widely used marker for keratinocyte differentiation, is a major component of the cornified envelope and its expression is relevant to the PPARG gene, which plays an important role in adipocyte differentiation [38]. Some potential candidate genes near those SNPs were the ARL15, CPS1, VWC2L, and VAV3 genes. ARL15 regulates human adiponectin levels [39], which affects insulin sensitivity and glucose and lipid metabolism [40]. CPS1-deficient hepatocytes can cause steatosis and glycogenosis [41]. VWC2L and VAV3 regulate osteoclast activation and matrix mineralization [42, 43].

Only suggestive SNPs were associated with ADG. Nevertheless, we still selected the gene COL27A1 as a potential functional candidate. It generates collagen type XXVII, and therefore it is crucial in cartilage calcification [44]. Most of the other genes close to suggestive SNPs played significant roles in nervous signal transduction and regulation. To evaluate the potential functional role of regions around associated SNPs with corresponding traits in our population, gene ontology (GO) information for each closest gene was collected (S2 Table). The nearest genes associated with the D100 trait primarily participate in the phosphorus metabolic process and neuron system. Most of the nearest genes associated with the FCR trait join the phosphorus metabolic process and nucleotide binding. It is essential to further investigate their effect on the phenotype to identify new pathways and mechanisms.

The results of our study agree, in part, with previous QTL mapping and GWAS studies. For example, previous publication reported a QTL associated with FCR on SSC16 located at 32~38 Mb with the peak at 35 Mb, is similar to our result, which showed that one haplotype block associated with FCR on SSC16 from 34.9 to 38.9 Mb. Furthermore, 12 significant SNPs for FCR located on SSC16 were detected in both studies [13]. Two QTLs for ADG were reported on SSC8 from 124.2 to 139.0 Mb and on SSC10 from 0.7 to 61.2 Mb respectively [45], this was confirmed by our study, two haplotype blocks associated with D100 and ADG were identified on SSC8 from 129.4 to 129.6 Mb and on SSC10 from 59.0 to 59.2 Mb respectively. Because of high correlation between ADG and D100, these two traits were also found to be associated with 9 same suggestive SNPs in our study. In conclusion, most of our results agree with previous research and further narrows down the ranges [13, 45, 46]. However, there is also some inconsistent results found in our study, for example, one haplotype block associated with FCR was found on SSC15 from 128.9 to 129.3 Mb, which is different from other reports [16]. We speculated that the different population background might lead to the disagreement. ADG is a complex trait with high heritability; however there are no significant SNPs were discovered to be associated with ADG. Similar results were reported in other studies [47, 48], which also indicated no or few significant SNPs were found to be associated with high heritability traits. The discrepancy might be caused by the following reasons. One is large number of causal variants with smaller effect are difficult to identify statistically [47–49]. Another reason is rarer variants with large effect do not exist in current commercial SNP chip [47–49]; Moreover, our investigated Duroc population has similar genetic background because of breeding purpose, more rare SNPs and monomorphic SNPs were filtered out, which also can lead to no significant SNPs found.

All candidate genes have been selected given their function and physical location near significant or suggestive SNPs associated with a production trait (D100, FCR, and ADG). To prove causality, future research must include gene sequencing and identification of all mutations, further statistical association testing, and cell experiments comparing molecular activities between mutant and normal cell lines. A more practical animal breeding aspect of our research could be weighting SNPs in genomic selection according to their relative additive effects on production traits.

## Supporting Information

S1 Fig Cumulative contribution rate of top ten principle components. PC1: First Principle component; PC2-PC10: First two top principle components- First ten top principle components;
(EPS)

S1 Table Chromosome-wide significant SNPs and nearest genes for three traits. (XLSX)

S2 Table Go ontology (GO) results for nearest genes. (XLSX)
